# Systolic anterior motion due to morphology changes in constrictive pericarditis

**DOI:** 10.1093/icvts/ivad146

**Published:** 2023-09-26

**Authors:** Yu Kumagai, Hiroyuki Nakajima, Tomomi Nakajima, Akihiro Yoshitake

**Affiliations:** Department of Cardiovascular Surgery, Saitama Medical University, International Medical Center, Saitama, Japan; Department of Cardiovascular Surgery, Saitama Medical University, International Medical Center, Saitama, Japan; Department of Cardiovascular Surgery, Saitama Medical University, International Medical Center, Saitama, Japan; Department of Cardiovascular Surgery, Saitama Medical University, International Medical Center, Saitama, Japan

**Keywords:** Constrictive pericarditis, Pericardiectomy, Systolic anterior motion, Left ventricular outflow tract obstruction, End-stage chronic kidney

## Abstract

Systolic anterior motion (SAM) can be caused by multifactorial mechanisms, including structural, morphological and functional factors. We report an unusual case of a 76-year-old woman presenting with SAM associated with constrictive pericarditis. Echocardiography showed no septal hypertrophy but SAM and left ventricular outflow tract obstruction and moderate mitral regurgitation. The restoration of diastolic function after complete pericardiectomy successfully eliminated it.

## CASE

**Figure 1:**
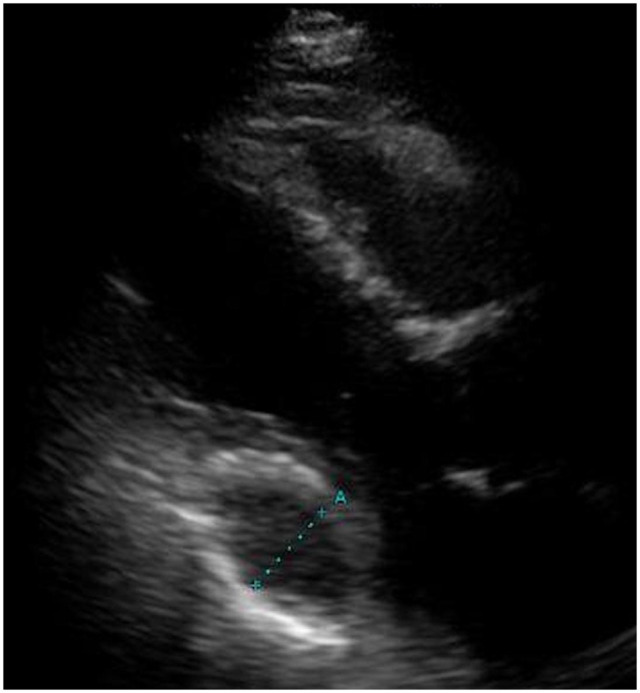
On transthoracic echocardiography, the mitral leaflets appeared compressed by the pericardial mass located posterior to the left ventricle and deviated anteriorly.

A 74-year-old woman, with end-stage renal failure caused by IgA nephropathy, underwent haemodialysis for 2 years. She was referred to our hospital because her systolic blood pressure was below 80 mmHg during haemodialysis and exertional dyspnoea. Transthoracic echocardiography (TTE) revealed a narrowed left ventricle with 36-mm diastolic dimension. The ejection fraction of the left ventricle was 82%. A thickened pericardium of 20 mm was seen around the heart maximal behind the mitral annulus, as a finding of constrictive pericarditis (Fig. 1). The anterior leaflet of the mitral valve was elongated to 31 mm, with systolic anterior motion (SAM) and left ventricular outflow tract obstruction (LVOTO) with a peak pressure gradient of 60 mmHg and moderate mitral regurgitation (Video 1). The right ventricular outflow tract diameter was 14.7 mm; the diameter at the base of right ventricle (RV) at four-chamber view was 20.8 mm. No septal hypertrophy was observed. On computed tomography, the pericardium was not calcified, but a severely thickened pericardium surrounded the atrioventricular groove. During heart catheterization procedure, the RV pressure waveform displayed a dip and plateau, indicating elevated RV pressure (39/10 mmHg), as characteristic findings of constrictive pericarditis, whereas the aortic pressure was low (86/50 mmHg). She was diagnosed with constrictive pericarditis complicated by LVOTO caused by SAM and treated surgically.

Through a median sternotomy, the pericardium was incised and dissected. The thickened pericardium consisted of a capsule of fibrous tissue (like an old haematoma). Pericardial tissues were fully resected around the ventricles without cardiopulmonary bypass (Video 2).

The patient was extubated on postoperative day 1. From days 1 to 4, continuous renal replacement therapy was administered, and on day 5, the treatment transitioned to haemodialysis. On day 7, TTE showed a considerable alleviation of SAM, with a peak pressure gradient of 7 mmHg. Additionally, the previously present mitral regurgitation had completely resolved. The systolic blood pressure during haemodialysis rose above 110 mmHg, and subsequently, the associated exertional dyspnoea improved, and the patient was discharged on day 15.

**Figure 2:**
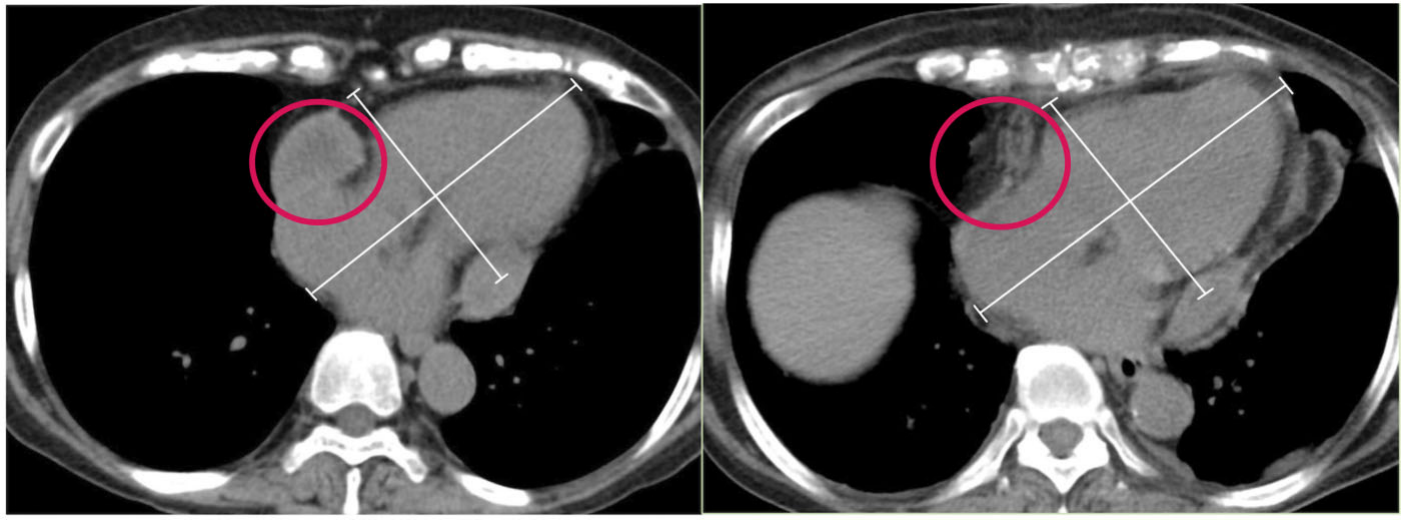
(Left) Computed tomography shows circumferential thickening of the pericardium. The heart measured 131 mm × 93 mm. (Right) The thickened pericardium was removed, resulting in the enlargement of the heart (circle). The heart dilated to 143 mm × 102 mm.

On the 6-month follow-up TTE, the left ventricular diastolic dimension was normalized to 43 mm, and SAM completely disappeared, with a peak pressure gradient of 3.2 mmHg (Video 3). right ventricular outflow tract diameter was 16.8 mm; the diameter at the base of RV at four-chamber view was 37.9 mm. All values improved postoperation. On computed tomography, the size of the short- and long-axis diameter of the heart was dilated (Fig. 2).

## DISCUSSION

Constrictive pericarditis represents cardiac function impairment by restricting the ventricular filling during the diastolic phase because of the thickened and calcified pericardium. The thick and calcified pericardium causes small ventricles or narrowing and tubular deformation of the right or left ventricle [1].

It rarely causes valvular dysfunction, whereas pericardiectomy could occasionally cause alteration of ventricular morphology and affect atrioventricular valve function. Tabucanon *et al.* [2] reported that 26.2% of patients who underwent pericardiectomy experienced worsening mitral regurgitation.

SAM is defined as the displacement of the mitral valve into the left ventricular outflow tract during systolic ejection, typically concomitant with septal hypertrophy. However, SAM could occur even without septal hypertrophy. Morphology factors contributing to SAM include long anterior or posterior leaflets, bulging septum, small left ventricle and anterior deviation of the papillary muscle [3]. Moreover, Abbas reported that the hyperdynamic state with inotropes and hypovolaemia due to diuretics caused transient SAM, even in a structurally normal ventricle [4].

Our patient had some potential contributing factors for SAM, other than septal hypertrophy. The anterior mitral leaflet was relatively long, and the patient had a small, tubular ventricular cavity and hyperdynamic state. Moreover, since the left ventricular cavity is smaller not only in the short axis but also in the long axis, elongation of the mitral valve leaflet is accentuated. Furthermore, the thickened pericardium exerted external pressure on the mitral valve, pushing it forward from the posterior wall side, and consequently LVOTO. The mechanism of SAM appeared to be of the length of the anterior mitral leaflet, shortening in the short and long axes and thickening of the posterior wall due to constrictive pericarditis. Pericardiectomy increased the distance between the papillary muscle and mitral annulus in the long axis and the left ventricular dimension in the short axis. Ultimately, the factors leading to SAM were successfully addressed without requiring any intracardiac manoeuvres.

## CONCLUSIONS

Constrictive pericarditis should be recognized as an unusual cause of SAM. Complete pericardiectomy resulted in the long- and short-axis dilation of the left ventricular cavity and eliminated SAM, mitral regurgitation and LVOTO.

## CONSENT

Consent of the patient was obtained for this study.

**Conflict of interest:** none declared.

## Data Availability

The data underlying this article cannot be shared publicly for the privacy of individuals that participated in the report. The data will be shared on reasonable request to the Corresponding author.
